# Stabilization of SETD3 by deubiquitinase USP27 enhances cell proliferation and hepatocellular carcinoma progression

**DOI:** 10.1007/s00018-021-04118-9

**Published:** 2022-01-12

**Authors:** Tingting Zou, Yang Wang, Ling Dong, Tiantian Che, Huakan Zhao, Xiaohua Yan, Zhenghong Lin

**Affiliations:** 1 School of Life Sciences, Chongqing University, Chongqing, 401331 People’s Republic of China; 2 Laboratory Research Center, The First Affiliated Hospital of Chongqing Medical University, 1st You Yi Road, Yuzhong District, Chongqing, 400016 People’s Republic of China; 3 Institute of Cancer, Xinqiao Hospital, Third Military Medical University, Chongqing, 400038 People’s Republic of China; 4 Department of Biochemistry and Molecular Biology, School of Basic Medical Sciences, Nanchang University, Nanchang, 330006 Jiangxi People’s Republic of China

**Keywords:** SETD3, USP27, Deubiquitination, Hepatocellular carcinoma, Cell proliferation

## Abstract

The histone methyltransferase SETD3 plays critical roles in various biological events, and its dysregulation is often associated with human diseases including cancer. However, the underlying regulatory mechanism remains elusive. Here, we reported that ubiquitin-specific peptidase 27 (USP27) promotes tumor cell growth by specifically interacting with SETD3, negatively regulating its ubiquitination, and enhancing its stability. Inhibition of USP27 expression led to the downregulation of SETD3 protein level, the blockade of the cell proliferation and tumorigenesis of hepatocellular carcinoma (HCC) cells. In addition, we found that USP27 and SETD3 expression is positively correlated in HCC tissues. Notably, higher expression of USP27 and SETD3 predicts a worse survival in HCC patients. Collectively, these data elucidated that a USP27-dependent mechanism controls SETD3 protein levels and facilitates its oncogenic role in liver tumorigenesis.

**Supplementary Information:**

The online version contains supplementary material available at 10.1007/s00018-021-04118-9.

## Introduction

SET domain-containing 3 (SETD3) is a member of the protein lysine methyltransferase (PKMT) family, which catalyzes the addition of methyl group to lysine residues [1]. Among SET domain lysine methyltransferases, SETD3 clusters with SETD4 and SETD6, which are unique in that they share a Rubisco-substrate binding (RSB) domain homologous to large subunit methyltransferase (LSMT) of plant Rubisco [2, 3]. It was first identified in Zebrafish by Kim et al. and they reported that SETD3 methylate histone H3 at Lys-36 (H3K36) and play pivotal roles in cell death and cell cycle regulation [4]. In addition, it has been reported that SETD3 is highly expressed in muscular tissues. SETD3 was recruited to the myogenin gene promoter along with MyoD where it activated transcription, thereby promoting mouse muscle cell differentiation. On the contrary, muscle cell differentiation was impeded by knockdown of SETD3 [5]. In the study of Wilkinson et al., SETD3 was identified as the physiologic actin histidine 73 methyltransferase, preventing primary dystocia [2]. Apart from that, SETD3 is potentially involved in DNA replication and DNA-damage-induced apoptosis [6, 7]. Moreover, Levy’s team demonstrated that human SETD3 (hSETD3) can methylate the transcriptional factor FoxM1 and regulate VEGF expression under low oxygen levels [1].

Many methyltransferases have critical roles in various biological processes, and their dysregulation is often associated with cancer. Accordingly, recent studies have also linked the abnormal expression of SETD3 to carcinogenesis [8, 9]. For instance, higher expression level of SETD3 was found in renal cell tumors compared to normal renal tissues, and high expression of SETD3 was inversely correlated with disease-free survival [10]. Similarly, the overexpression of SETD3, which lacks the SET domain, displays oncogenic properties in lymphoma [9]. Hassan et al. showed that SETD3 can act as a prognostic marker in breast cancer patients and modulates the viability and invasion of breast cancer cells. The inhibition of SETD3 expression blocks the viability and invasiveness of breast cancer cells [11]. Furthermore, another study indicated that SETD3 level is positively correlated with cell proliferation of liver cancer cells and liver tumorigenesis in a xenograft mouse model [12]. Thus, these data possibly suggested a pro-tumorigenic role of SETD3. However, how SETD3 expression is regulated remains unknown.

Ubiquitin-mediated proteolysis is one of the most important pathways to regulate the abundance of intracellular proteins. It has been shown that E3 ubiquitin ligase SCF^FBXW7β^ can interact with phosphorylated SETD3 which is mediated by GSK3β, promoting its ubiquitination and degradation. Depletion of FBXW7β or GSK3β increased SETD3 protein levels, thereby facilitating tumor growth [12]. Ubiquitination is a reversible modification. Deubiquitinating enzymes (DUBs) can remove the ubiquitin chain from the substrate proteins, reducing proteasomal recognition and thereby stabilizing the proteins [13, 14]. Ubiquitin-specific peptidase 27 (USP27, as known as USP27x) is a DUB and a member of the cysteine protease family, which contains an N-terminus and a ubiquitin-specific peptidase domain at its C-terminus [15]. A previous study suggested that USP27, together with USP22 and USP51, regulates mono-ubiquitination of histone H2B [16]. Moreover, USP27 deubiquitinates and stabilizes the BH3-only protein Bim, subsequently enhancing apoptosis [14]. A recent study indicated that USP27 can participate in cell cycle progression by regulating the abundance of cyclin E [15]. However, whether USP27 exerts biological functions through other substrate(s) remains largely unknown.

Herein, we unraveled that USP27 is able to interact with SETD3, promoting its deubiquitination and stabilization. Knockdown of USP27 by short hairpin RNA (shRNA) accelerated the degradation of SETD3 and blocked cell proliferation. In addition, we found that USP27 and SETD3 expression are positively correlated in human hepatocellular carcinoma (HCC), and tumor patients with lower expression levels of SETD3 and USP27 have a longer five-year survival. Our findings in this study could better understand the roles and molecular mechanisms of USP27–SETD3 axis in cancer development, allowing for contributing to new strategy for cancer treatment.

## Results

### Library screening identified USP27 as a SETD3 interactor

**Fig. 1:**
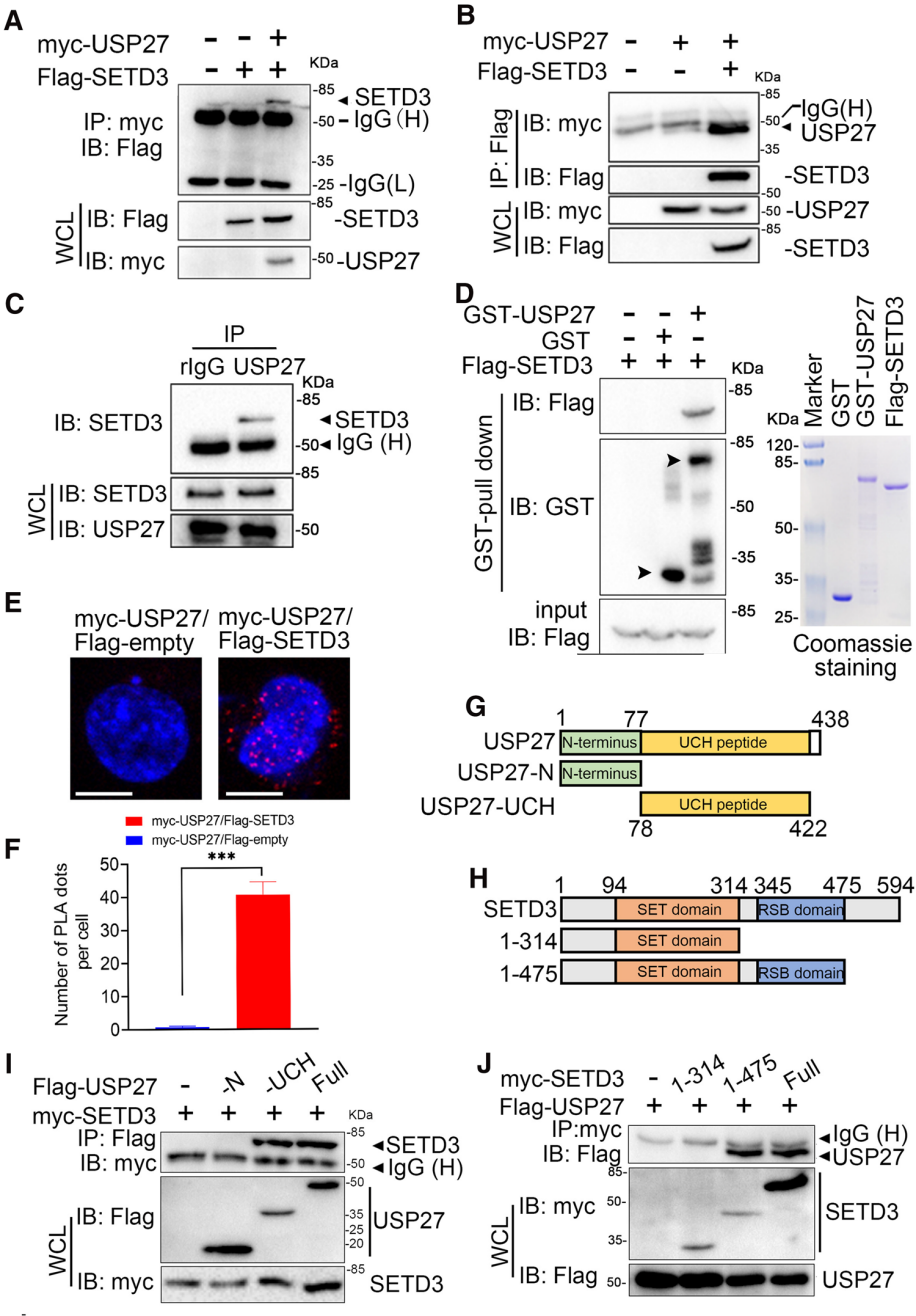
USP27 interacts with SETD3. **A**, **B** SETD3 or USP27 plasmids were transfected alone or together into 293 T cells and their interaction was determined by immunoprecipitation and western blot. **C** The endogenous interaction of USP27 and SETD3 was tested in Hep3B cells. **D** The interaction between USP27 and SETD3 was detected by GST pull-down assay in vitro. **E** Proximity ligation assay (PLA) indicating sites of direct protein–protein interaction between myc-USP27 and Flag-SETD3 in 293 T cells (red signal). Cells transfected with myc-USP27 and empty Flag vector served as a control. DAPI (blue signal) was used to counterstain the nuclei. DAPI: 4, 6-diamidino-2-phenylindole. Bar, 10 μm. **F** Quantification of USP27–SETD3 PLA analysis. Data are mean ± SEM of three different fields. ***p < 0.001. **G** Schematic picture of full-length USP27 and its mutants. **H** SETD3 and its domains. It contains a N-terminal, SET domain and RSB domain. **I** SETD3 interactions with USP27 and its mutants were tested as indicated. **J** The interactions of SETD3 and its mutants with USP27 protein were examined

To determine the molecular mechanisms underlying the aberrant protein expression of SETD3 in liver tumorigenesis [12], we used a DUB library [15] to screen SETD3-interacting protein, which might be responsible for its dysregulation. Briefly, we transfected each of Flag-tagged DUBs with myc-tagged SETD3 constructs into HEK293T (293 T) cells, and 48 h later we tested the interactions between SETD3 and each DUB by co-immunoprecipitation (Co-IP) and western blotting (WB). As seen in supplementary Fig. S1, USP27 was identified as SETD3 interaction partner among DUBs. To further confirm that the interaction between SETD3 and USP27 was specific, we transfected them alone or together into 293 T cells and evaluated their interaction by immunoprecipitation and western blot. As shown in Fig. 1A and B, SETD3 could be pulled down by USP27, and conversely, SETD3 was also able to pull-down USP27, suggesting that USP27 are true SETD3 interaction partner. Similarly, we examined whether endogenous SETD3 interacts with USP27 in Hep3B cells. As expected, SETD3 was detected in the anti-USP27 but not normal rabbit IgG immunoprecipitates (Fig. 1C). In addition, this direct interaction was confirmed by GST pull-down assay in vitro (Fig. 1D). Next, in situ proximity ligation assay (PLA), which enables detecting the protein–protein interactions directly within the cell, further proved that USP27 interacts with SETD3 (Fig. 1E, F). Finally, we generated truncated mutants to determine the interaction region between USP27 and SETD3 (Fig. 1G, H). USP27 contains an N-terminus and C-terminal UCH domain. The C-terminal UCH domain of USP27 is required for its interaction with SETD3 because deletion of this region completely abolished their interaction. In contrast, expression of the C-terminal 78–422 fragment alone is sufficient for their interaction (Fig. 1I). Furthermore, truncated mutation analyses showed that the RSB domain of SETD3 is required for its interaction with USP27 (Fig. 1J). Taken together, these data suggested that USP27 interacts with SETD3.

### USP27 inhibits the K48-linkage poly-ubiquitination of SETD3

**Fig. 2:**
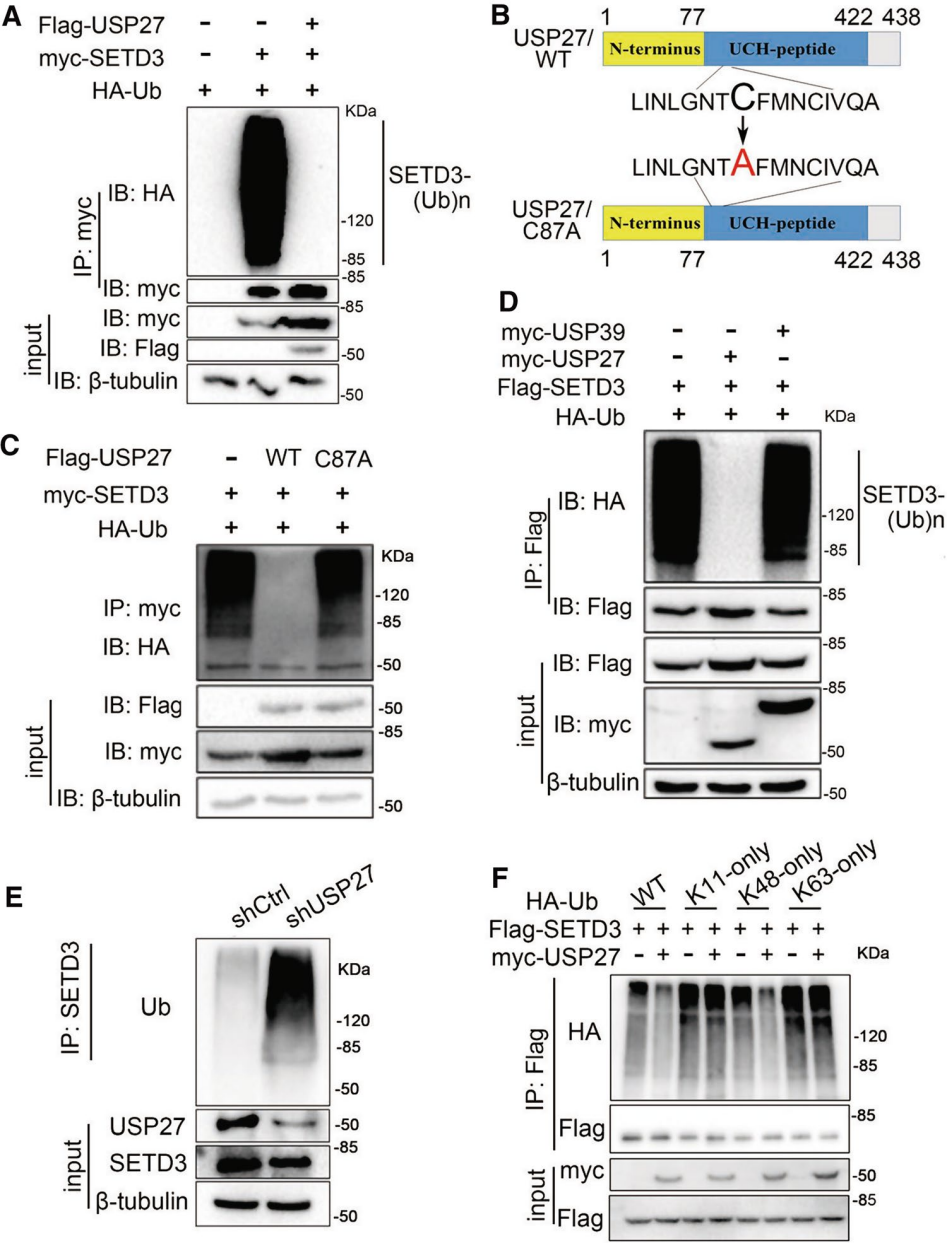
USP27 inhibits the K48-linkage poly-ubiquitination of SETD3. **A** HA-Ubiquitin, Flag-USP27, and myc-SETD3 plasmids were transfected alone or cotransfected into 293 T cells. Forty hours later, cells were harvested and the ubiquitination of SETD3 was detected by immunoprecipitation and western blot. The expression of SETD3 and USP27 were confirmed by western blot. **B** Schematic showing of wide type (WT) USP27 and its enzyme-inactive mutants (C87A). The cysteine (C) residue in position 87 in the UCH domains was replaced by alanine (**A**). **C** The effects of WT USP27 and its enzyme-inactive mutants (C87A) on SETD3 ubiquitination were analyzed as in **A**. **D** HA-Ubiquitin and Flag-SETD3 expression plasmids were cotransfected with myc-USP27, or myc-USP39 into 293 T cells and SETD3 ubiquitination was performed as in **A**. **E** The ubiquitination of endogenous SETD3 in Hep3B-control and stable knockdown USP27 cells was analyzed by immunoprecipitation with anti-SETD3 antibody and western blot with anti-ubiquitin antibody. β-tubulin are used as loading control. **F** Western blot analysis of ubiquitination level of Flag-SETD3 in 293 T cells cotransfected with plasmids expressing Flag-SETD3, myc-USP27, and HA-tagged WT ub or HA-tagged mutant Ub (K11 only, K48 only, K63 only). Cells were treated with proteasome inhibitors MG132 (50 μM) for 3 h before harvested

As USP27 is a DUB that might protect its substrate from proteasome-mediated degradation, we next determined whether this DUB removes ubiquitin from SETD3. SETD3 and ubiquitin plasmids were transfected into 293 T cells with or without USP27 addition. As shown in Fig. 2A, the polyubiquitin chains linked to SETD3 was completely removed in the presence of USP27 expression. Next, we generated an enzyme-inactive mutant of USP27 [15] (Fig. 2B) and assessed the effect of the mutation on SETD3 ubiquitination. As seen in Fig. 2C, an enzyme-inactive mutant of USP27 (C87A) failed to abolish the ubiquitination of SETD3 protein compared with the wild-type (WT) USP27, implying that the DUB activity is required for USP27 to remove ubiquitin from SETD3. Additionally, USP27-mediated SETD3 deubiquitination is pretty specific, since USP39, which was recently shown to be a CHK2-specific DUB [17], does not significantly affect SETD3 deubiquitination (Fig. 2D). Moreover, knockdown of USP27 expression resulted in elevated SETD3 ubiquitination compared with nonspecific short hairpin RNA (shRNA) control (Fig. 2E). Commonly, polyubiquitin chains with conjugation to the lysine 48 site (K48) or K11 site lead to 26S proteasome-mediated degradation of the labeled substrate, whereas K63-linked chains act a significant role in cellular signal transduction [18, 19]. Therefore, to determine which type of ubiquitin linkage of polyubiquitin chains on SETD3 is regulated by USP27, we used ubiquitin mutants in which only one Lysine residue at position 11 (K11O), 48 (K48O) or 63(K63O) was available for ubiquitination. As shown in Fig. 2F, USP27 overexpression reduced K48-linked ubiquitination of SETD3, but had no effect on K11- or K63-linked ubiquitination of SETD3. Collectively, these results suggested that USP27 is a true DUB for SETD3, and removed K48-linked polyubiquitin chains of SETD3 in a DUB activity-dependent manner.

### SETD3 protein stability is regulated by USP27

**Fig. 3:**
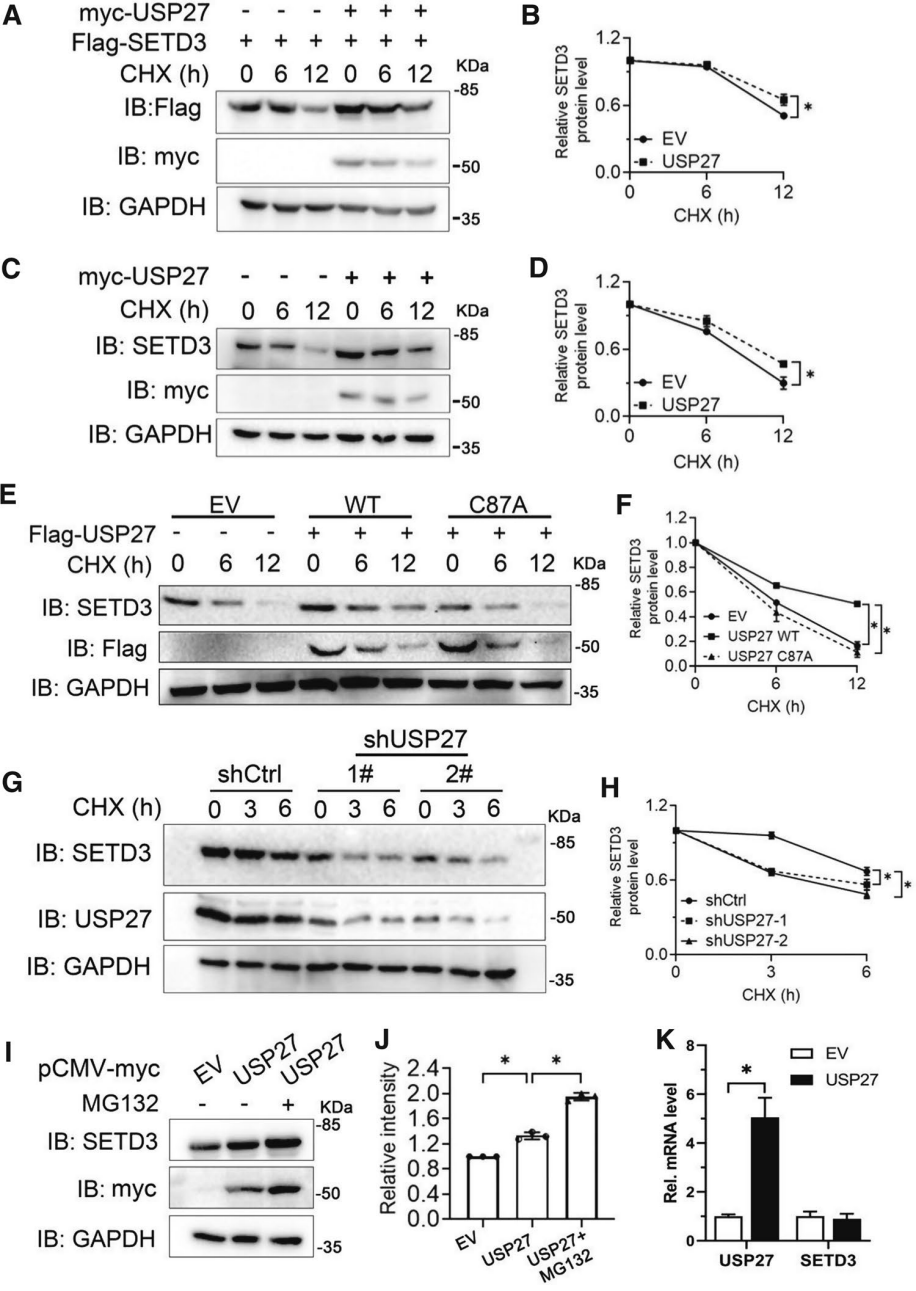
USP27 promotes SETD3 stabilization. **A**, **B** 293 T cells were transfected with SETD3 plasmids and empty vectors (EV) or USP27 plasmids and two days later were treated with 100 μg/ml cycloheximide (CHX) for indicated time and harvested. The expression of SETD3, USP27, and GAPDH were analyzed by western blot (**A**) and quantified (**B**). **C**, **D** 293 T cells were transfected with empty vectors or USP27 plasmids and the endogenous protein stability of SETD3 was examined as in **A**. **E**, **F** Empty vector, USP27 or USP27/CA mutant plasmids were transfected into 293 T cells. SETD3 protein stability in the transiently transfected 293 T cells was examined by western blot (**E**) and quantified (**F**). **G**, **H** SETD3 protein expression was analyzed in control or USP27 knockdown Hep3B cells. The expression of SETD3 and USP27 was determined by western blot (**G**) and quantified (**H**). **I**, **J** Hep3B cells were transfected with empty vector or USP27 plasmids as indicated. Forty hours later, cells were treated with or without MG132 (50 μM) for three hours and then cells were harvested. SETD3 and USP27 expression were examined by western blot (**I**) and relative intensity of SETD3 was shown (**J**). **K** Hep3B cells were transfected with empty vector or USP27 plasmids. Forty hours later, the mRNA levels of SETD3 and USP27 were determined by RT-qPCR. In **B**, **D**, **F**, **H**, and **J**, the error bars represent data from three independent experiments (mean ± SEM). *p < 0.05

DUBs can remove ubiquitin from substrates and protects target protein from 26S proteasome-mediated degradation [20]. Our previous finding has demonstrated that USP27 was able to promote the deubiquitination of SETD3 at K48 site. To examine whether this post-translational modification facilitates the stability of SETD3, the SETD3 expression constructs were cotransfected with empty vector (EV) or USP27 plasmids into 293 T cells and tested the protein level of SETD3 under the treatment of cycloheximide (CHX) at different time. As shown in Fig. 3A and B, SETD3 protein expression is significantly upregulated by cotransfection of USP27 and the half-life of the SETD3 protein is dramatically extended by USP27. Furthermore, ectopic expression of USP27 upregulated endogenous SETD3 protein levels (Fig. 3C and D). Importantly, the catalytically inactive USP27/CA mutant failed to protect SETD3 from degradation (Fig. 3E and F). To further confirm our notion that USP27 regulates SETD3 stability, we knocked down USP27 expression in Hep3B cells and found that the endogenous SETD3 protein levels decreased more rapidly than control (Fig. 3G and H). Moreover, we found that treatment with MG132, a proteasome-specific inhibitor, could protect SETD3 from degradation (Fig. 3I, J), suggesting that SETD3 protein degradation occurs through the proteasome pathway. Finally, no obvious change in the mRNA expression levels of SETD3 was observed in the Hep3B cells showing USP27 overexpression (Fig. 3K), suggesting that the regulation of SETD3 protein degradation by USP27 might occur at the post-transcriptional level. In conclusion, our data suggested that USP27 could positively regulate SETD3 protein stability.

### SETD3 reverses the USP27 knockdown mediated blockade of cell proliferation

**Fig. 4:**
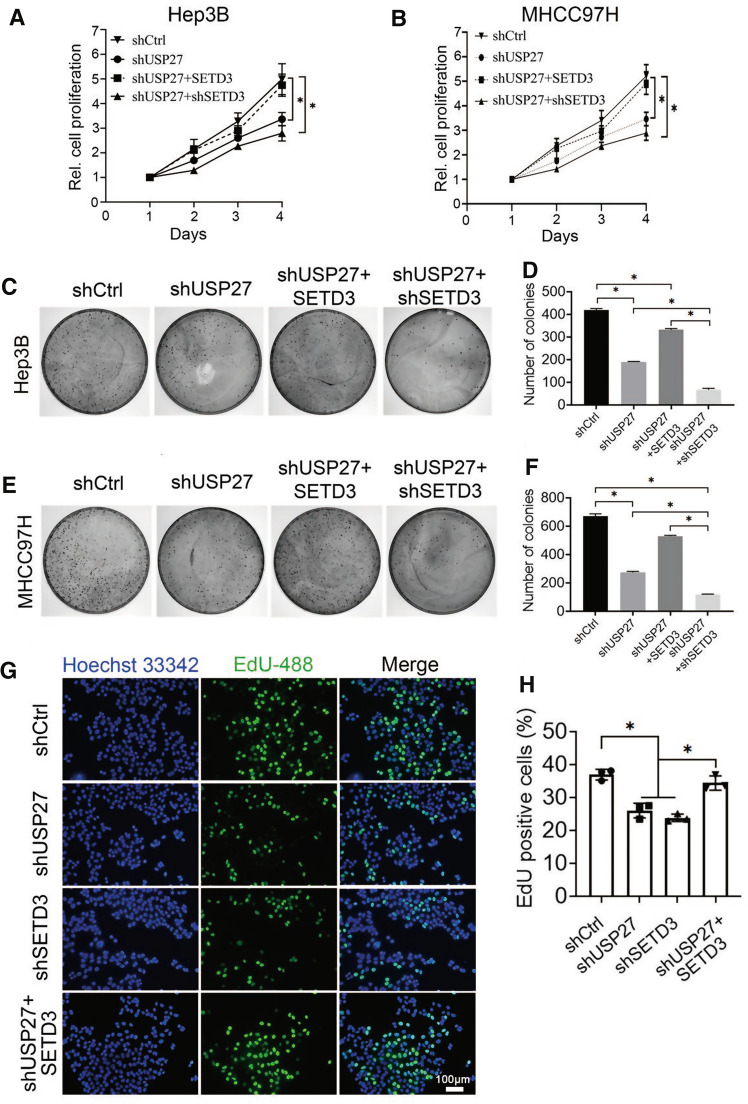
Suppression of USP27 expression blocked cell proliferation and delayed cell growth. **A**, **B** The proliferation of Hep3B (**A**) or MHCC97H (**B**) cells stably expressing control vector, shUSP27, shUSP27 + SETD3, and shUSP27 + shSETD3 were examined by CCK-8 assay. Data are represented as mean ± SEM of three independent experiments. *p < 0.05. **C**, **F** Colony formation assay was performed to measure the cell proliferation capacity of Hep3B or MHCC97H cells stably expressing the indicated plasmids. Images are the result of one representative experiment. Data are represented as mean ± SEM of three independent experiments. *p < 0.05. **G**, **H** Hep3B cells were incubated with EdU for 2 h. A fluorescence microscope was used to detect EdU, and Image J software was used to quantified (**H**). Data are represented as mean ± SEM of three independent experiments. *p < 0.05

SETD3 plays an important role in cancer development and is associated with several kinds of cancers [9–12]. Since USP27 could deubiquitinate and stabilize SETD3, it might promote cell proliferation and migration by regulating SETD3 protein levels. To test this hypothesis, we first assessed the biological role of USP27 in HCC by investigating the effects of USP27 knockdown on the viability and colony formation of Hep3B and MHCC97H cancer cells (Fig. 4). As shown in Fig. 4A and B, USP27 knockdown markedly reduce the cell viability of Hep3B and MHCC97H cells. Introducing SETD3 into USP27 knockdown cells partially restored the malignant phenotype, while knockdown of both proteins significantly inhibited cell ability. Colony formation assay further confirmed that the stable knockdown of USP27 in either Hep3B or MHCC97H cells significantly inhibited colony formation (Fig. 4C–F). Moreover, 5-ethynyl-2’-deoxyuridine (EdU) revealed that USP27 or SETD3 knockdown was associated with a marked percentage reduction in EdU-labeled cells, which can be partially reversed by the addition of SETD3 expression in the USP27 knockdown cells (Fig. 4G, H). Similarly, the decreased proliferation was also confirmed by Ki67 immunofluorescence staining as the proportions of Ki67-positive proliferating cells were significantly lower in USP27 or SETD3 knockdown cells, while SETD3 overexpression could increase Ki67-positive proliferating cells in USP27-deficient cells (Supplementary Fig. S2). The increase of Ki67-positive cells also suggested that more G0 quiescent cells re-entered the cell cycle upon SETD3 overexpression, since Ki67 is only expressed in active stages but not in G0 quiescent phase of the cell cycle. These results also showed that the total size of G0/G1 phase cell portion was added upon USP27 or SETD3 knockdown, thereby suppressing cell cycle progression. Collectively, these findings suggested that USP27 and SETD3 are essential for cell proliferation.

### USP27 knockdown inhibits HCC cell growth in vivo

**Fig. 5:**
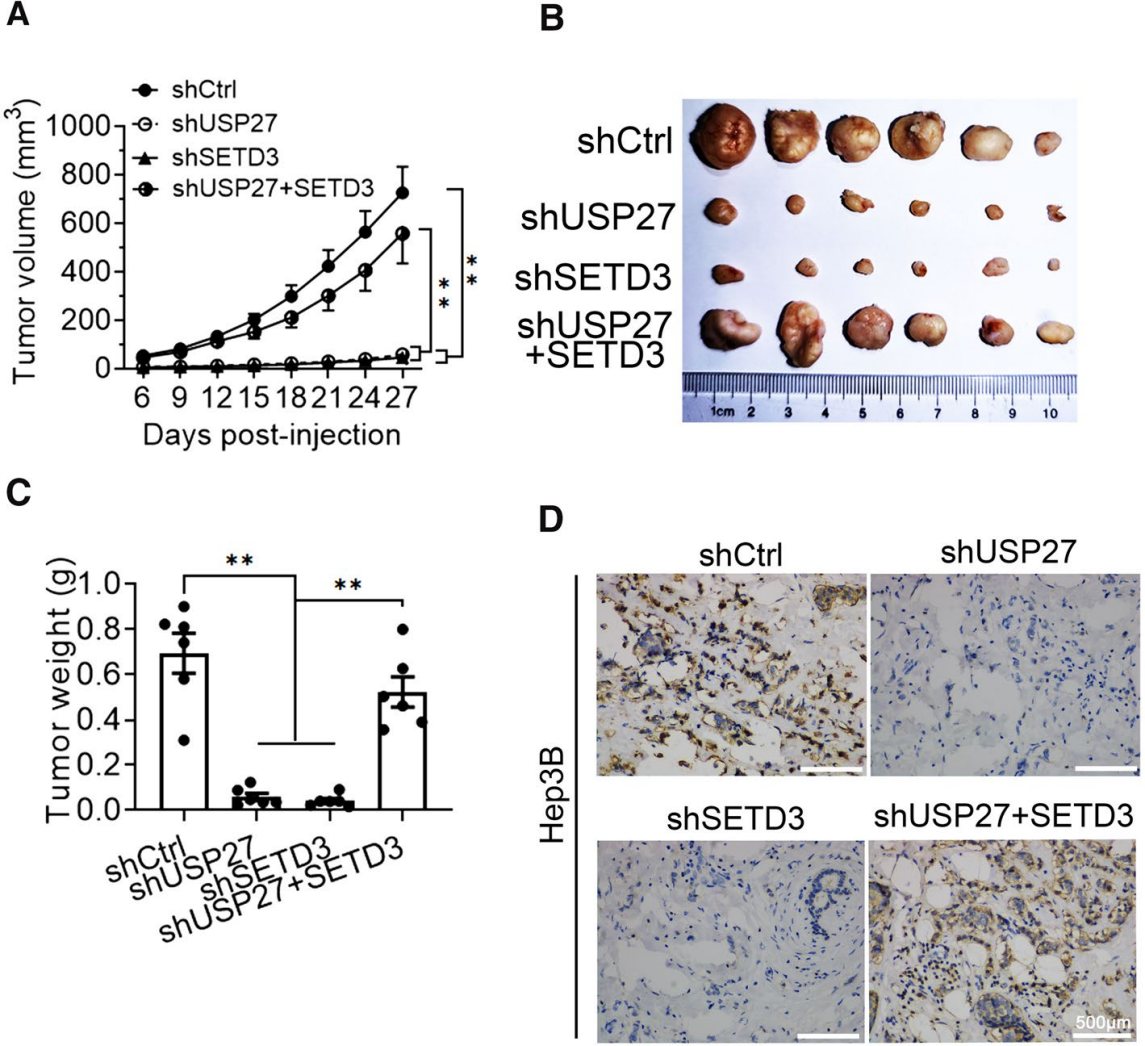
USP27 knockdown inhibited HCC cell growth in vivo. Tumorigenesis assay was performed by subcutaneous injection of USP27, SETD3 knockdown or USP27 knockdown overexpressing SETD3 cells into flanks of BALB/c nude mice. **A** Tumor volumes were measured at the indicated days post-injection. **B** and **C** Tumors were dissected, photographed (**B**) and weighted (**C**) at 27 days post-injection. **D** Ki67 staining showed that cell viability was decreased in the tumors of USP27 and SETD3 knockdown cell, while overexpression of SETD3 in USP27-deficient cells was able to restore cell viability. Data are presented as the mean ± SEM. n = 6 for each group. **p < 0.01

To assess the effect of USP27 and SETD3 on cell growth in vivo, we performed xenograft model by subcutaneous injection of Hep3B cell lines into the right flanks of nude mice. In the course of tumor development, the volumes of the tumors were measured every 3 days from day 6 after injection and the result showed that tumor growth was remarkably decreased in both USP27 and SETD3 knockdown cells (Fig. 5A). At 27 days post-injection, tumors were dissected, photographed (Fig. 5B) and weighted (Fig. 5C). Tumors generated by USP27 or SETD3 knockdown cells were remarkably smaller and lighter than the tumors of control cells, while SETD3 overexpression could restore tumor growth in USP27-deficient cells. In addition, cell viability was also decreased in the tumors of USP27 knockdown cells as determined by Ki67 staining (Fig. 5D). These results indicate that depletion of USP27 also inhibited HCC cell growth in vivo.

### Inhibition of USP27 expression impairs the pro-migratory ability of SETD3

**Fig. 6:**
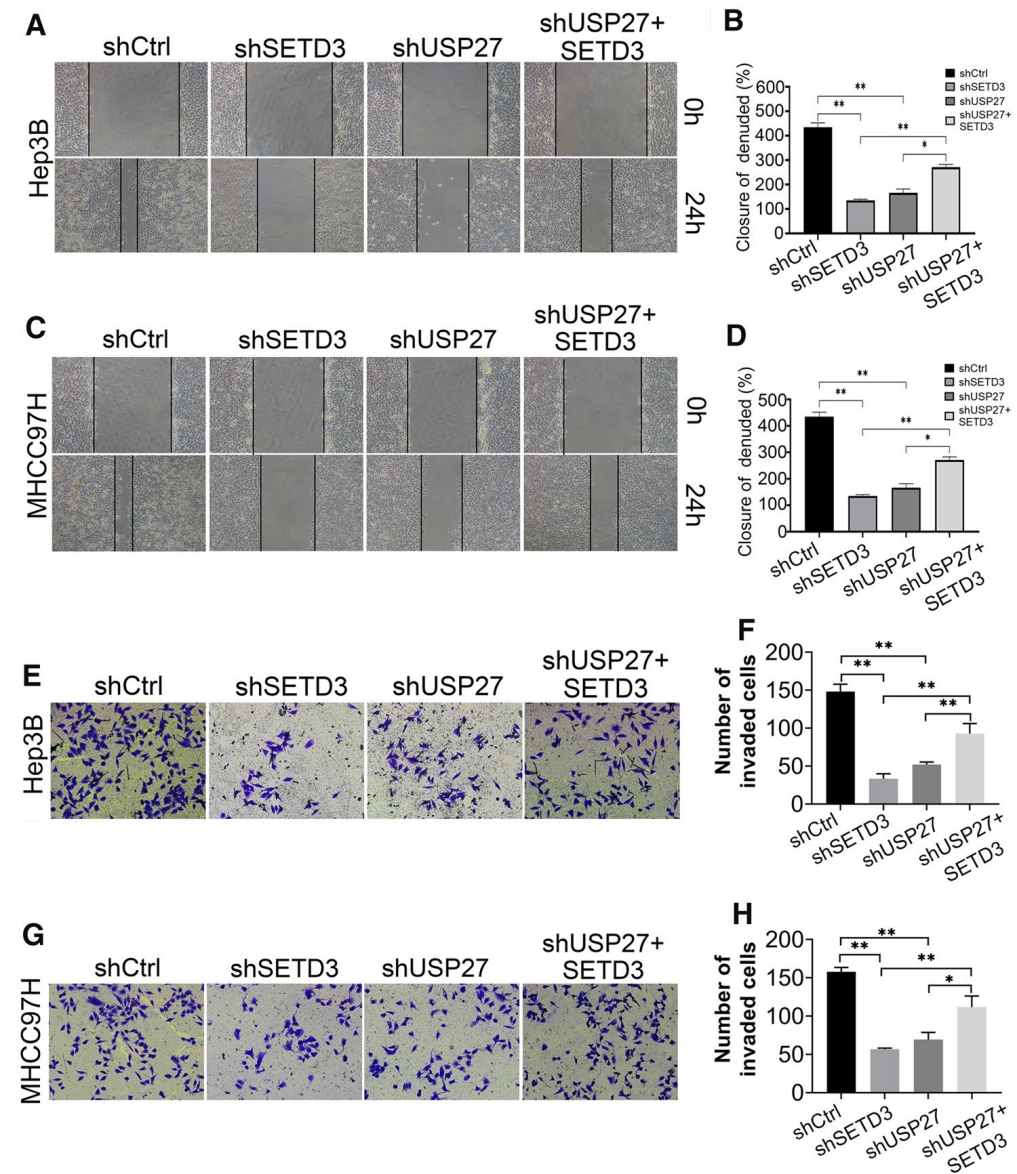
Inhibition of USP27 expression impairs the pro-migratory ability of SETD3 in hepatocellular carcinoma cells. **A–D** Wound-healing assays were performed to measure the migration of Hep3B or MHCC97H cells stably expressing the indicated single knockdown or a combination of plasmids. Cells were monitored within 24 h to evaluate the rate of migration into the scratched area. The wound edges are indicated by black lines (**A** and **C**). The wound closure percentage is shown (**B** and **D**). Data are represented as mean ± SEM of three independent experiments. *p < 0.05 and **p < 0.01. **E**, **H** Transwell assays were performed to measure the invasion ability of Hep3B or MHCC97H cells stably expressing the indicated single knockdown or a combination of plasmids. Data are represented as mean ± SEM of three independent experiments. *p < 0.05 and **p < 0.01

Since upregulation of SETD3 can promote liver tumorigenesis and cancer progression [12], it is possible that USP27 can also enhance metastatic phenotype by stabilizing SETD3. To explore the roles of the USP27–SETD3 axis in cell migration and invasion, we performed the wound-healing assay and the results showed that knockdown of SETD3 or USP27 significantly inhibited cell migration compared to that in the control cells, whereas introducing SETD3 into USP27 knockdown cells partially restored the metastatic phenotype (Fig. 6A-D). The Matrigel transwell invasion assays consistently showed similar results regarding the role of USP27 and SETD3 in the tumor invasion of Hep3B (Fig. 6E, F) and MHCC97H cells (Fig. 5G and H). Taken together, these data revealed that knockdown of USP27 or SETD3 prevents liver cancer cell migration and invasion.

### USP27 and SETD3 expression are upregulated in liver cancer samples

**Fig. 7:**
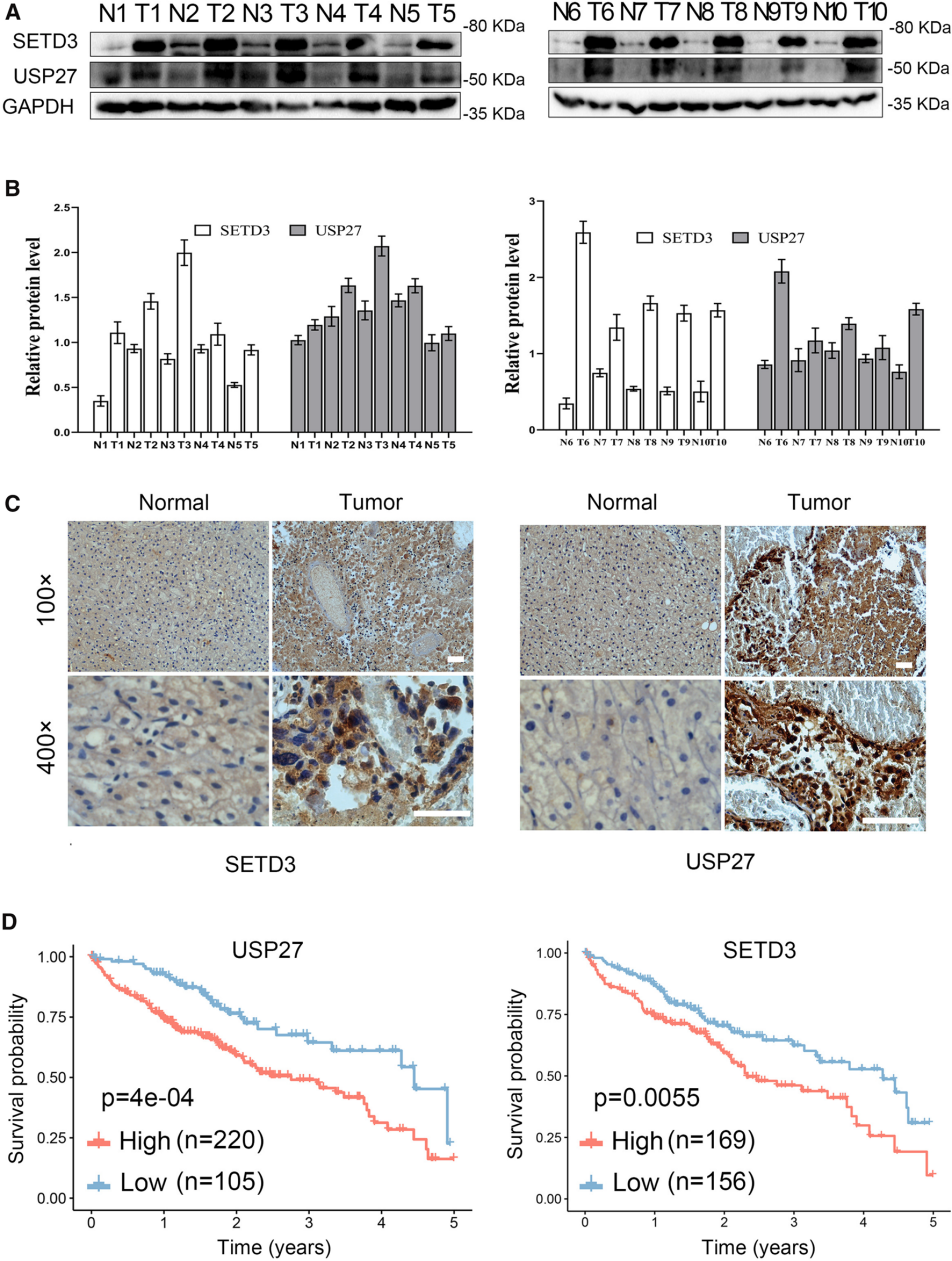
USP27 and SETD3 expression are upregulated in hepatocellular carcinoma tissues. **A** and **B** The expression of USP27 and SETD3 was measured in HCC and normal tissue (**A**) and quantified (**B**). **C** Representative images of immunohistochemical staining of USP27 and SETD3. Scale bars, 100 μm. **D** Kaplan–Meier survival curves and log-rank test were performed to compare the survival difference of the liver hepatocellular carcinoma (LIHC) patient samples. High expression is defined as greater than the average of normal tissues, and low expression is defined as less than or equal to the average of normal tissues. High USP27 or SETD3 expression represented in the red line, while low expression of USP27 or SETD3 is in blue. N, number of samples

The finding that USP27–SETD3 axis elevates the cell proliferation and migration prompted us to imagine that they might be upregulated in hepatocellular carcinoma. First, we examined the expression levels of USP27 and SETD3 in HCC tissues (n = 10) and matched adjacent normal liver tissues (n = 10) using western blot (Fig. 7A). As shown in Fig. 7B, USP27 and SETD3 protein levels were obviously upregulated and positively correlated. The relationship was further examined via the immunohistochemical staining of HCC tissues and normal tissues. As Fig. 7C showed, the expression level of SETD3 and USP27 were significantly upregulated in HCC tissues as opposed to matched normal liver tissues. It should be noted that data extracted from the TCGA database revealed that USP27 or SETD3 expression was notably higher in a variety of tumor types compared to matched TCGA normal tissues and GTEx data (Supplementary Fig. S3). Besides that, to estimate the prognostic impact of USP27 and SETD3 expression, Kaplan–Meier analysis of liver cancer patients from the TCGA repository (http://gdac.broadinstitute.org/) was performed using the R package. Patients were divided according to their mRNA expression level into high (expression above the average of normal tissues) and low (expression below or equal to the average of normal tissues) expressing groups, and survival was compared between the two groups. As shown in Fig. 7D, the 5-year survival rate was significantly reduced for patients with high expression of USP27 compared with those with low expression. Similar results were obtained in the analysis of SETD3.

Based on our results, we concluded that SETD3 is deubiquitinated by USP27 and its protein level is positively regulated by and correlated with USP27 expression. Upregulation of USP27 in hepatocellular carcinoma patients leads to elevated SETD3 expression and increased cell proliferation, invasion, migration and tumorigenesis.

## Discussion

**Fig. 8:**
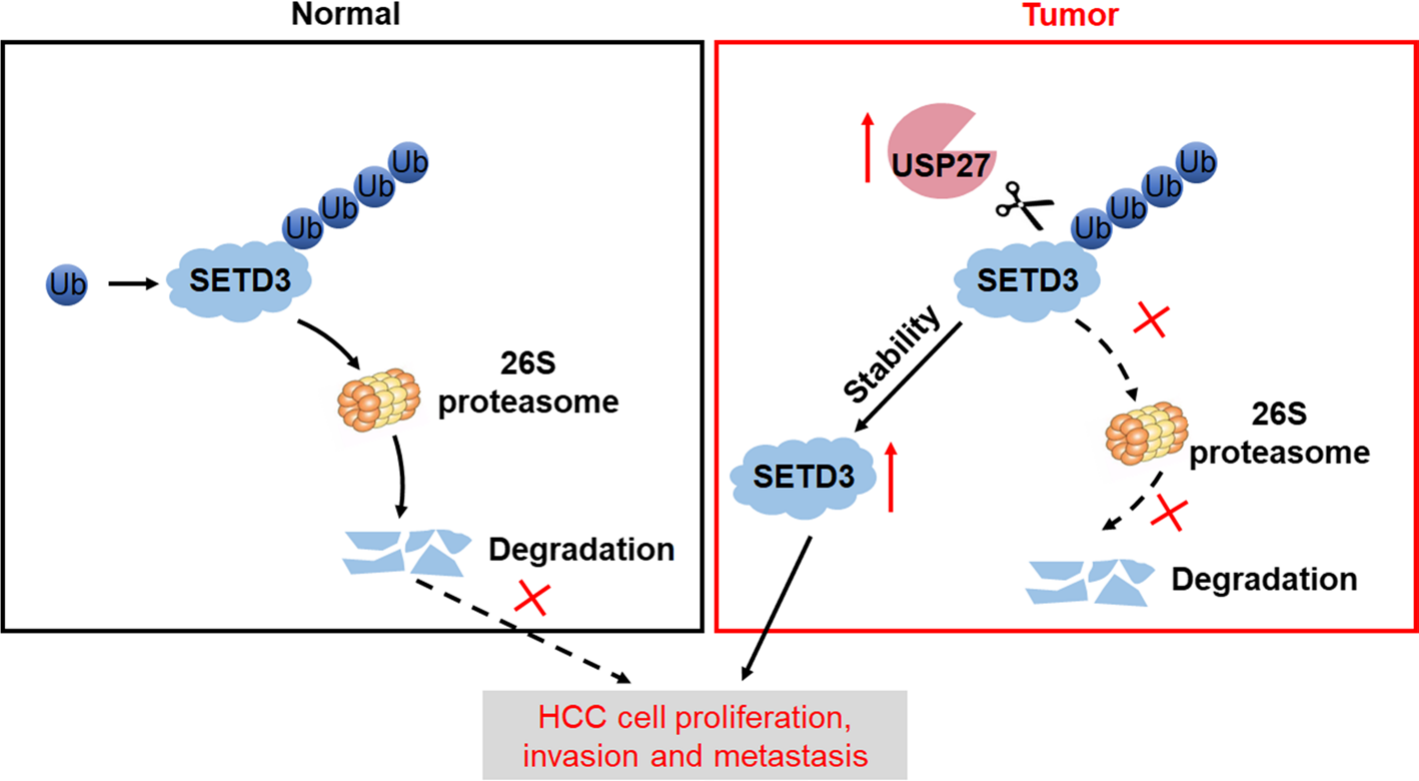
Schematic working model of SETD3 regulation by USP27. Upregulation of USP27 in HCC leads to stabilization of SETD3, thus promoting cell proliferation, tumorigenesis and cancer progression

In this study, we identified new regulator of SETD3 stability and function (Fig. 8). We found that SETD3 was deubiquitinated and stabilized by USP27. This DUB physically interacts with SETD3 and remove K48-linked polyubiquitin chains from SETD3 by their catalytic activities. Overexpression of USP27 stabilizes SETD3 and increases its half-life. Inhibition of USP27 resulted in significant decreased endogenous SETD3 levels, suggesting that USP27 is the true DUB for SETD3. Furthermore, USP27 or SETD3 knockdown inhibits cell proliferation, cell migration and tumorigenesis, while overexpression of SETD3 in USP27-deficient HCC cells could restore cell viability. These results suggested that deubiquitination regulates SETD3 functions by stabilizing it in vivo.

USP27 is a DUB and belongs to cysteine protease family. Previous studies have demonstrated that USP27 is involved in several biological processes. For example, Weber et al. found that wild-type USP27 can reduce the levels of Bim ubiquitination and stabilize Bim in response to the Raf‐ERK‐degradation signal [14]. In another study, USP27 was confirmed to regulate cell migration and chemoresistance via stabilizing Snail1 [21]. In addition, USP27 could regulate neuronal differentiation of stem cells in the developing mouse neocortex by deubiquitinating and stabilizing Hes1 [22]. There is also evidence that USP27 is also involved in immune regulation. Guo et al. demonstrated that USP27 contributes to innate immunity by removing K48-linked ubiquitination of cGAS [23]. These results indicate that USP27 can target different substrates to participate in the regulation of different signaling pathways. Therefore, identifying new substrate for USP27 will expand our new understanding of USP27 biological function.

SETD3 is a member of the protein family containing SET domain [24]. Recently, SETD3 was reported as the first and so-far the only known metazoan histidine methyltransferase that catalyzes actin histidine 73 (His73) methylation [2, 25]. In 2011, Kim et al. identified that Zebrafish SETD3 involves in transcription activation, and its overexpression in mammalian cells suppresses cell survival [4]. The same group further demonstrated that SETD3 is associated with skeletal muscle cell differentiation, and knockdown of SETD3 attenuated myoblast differentiation [5]. Interestingly, the muscle cell differentiation induced by SETD3 can be inhibited by miR-15b and miR-322 [26]. In addition, other studies also implied that SETD3 is associated with DNA replication or DNA repair [6], cancer proliferation and metastasis [11, 27]. Therefore, it is very important to explore how the protein expression level of SETD3 is regulated. About 80% of proteins are degraded by the ubiquitin–proteasome system (UPS) [28, 29]. Protein ubiquitylation regulates the stability and activity of certain target proteins that are involved in the pathogenesis of various cancers [30, 31]. A study reported in 2017 has validated the potential mechanism of a E3 ligase SCF^FBXW7^ by ubiquitinating SETD3 to regulate the liver cancer progression [12]. However, there is no report on the deubiquitinase of SETD3.

Here, we elucidated the dynamic regulation of SETD3 through the orchestrated post-translational modifications by a USP27-dependent pathway. We first identified USP27 as an interacting partner of SETD3 in Hep3B cells. USP27 was able to deubiquitinate and stabilize SETD3. Furthermore, evidence from in vitro and in vivo studies revealed that depletion of USP27 inhibited HCC cell proliferation, invasion, metastasis and tumorigenesis, and that overexpression of SETD3 rescued this phenotype. We also found that the levels of SETD3 and USP27 increased significantly in HCC than those in the relevant adjacent tissues. By using data available from the TCGA and the GTEx databases, we here showed the expression levels of USP27 and SETD3 are negatively correlated with the 5-year survival rate of HCC patients. Collectively, we revealed that USP27 exerts tumor-promoting action by modulating the USP27–SETD3 axis.

Studies have validated that USP22, the USP27 homologous protein, has similar effects with USP27 [16, 21, 22]. In this study, we did not determine whether USP22 can also interact with SETD3 and play a deubiquitination function. In previous studies, USP27 seemed to prefer to remove the K48-linked polyubiquitin chain of the substrates, thereby stabilizing proteins [23]. In this study, we also proved that USP27 can protect SETD3 from proteasome degradation by cleaving the polyubiquitin chain at K48. However, whether USP27 can cleave the polyubiquitin chains at other sites of SETD3 still needs to be further explored.

## Materials and methods

### Plasmids, antibodies and reagents

USP27, SETD3 and their mutants were cloned into pCMV-Flag2.0, pCMV-Myc (Addgene Company) or pSEB-3Flag vectors. USP27-C87A mutant was made using PCR-based site-directed mutagenesis method. All other constructs were generated using standard molecular cloning methods and were confirmed by DNA sequencing. Antibodies used for immunoprecipitation, immunoblotting, and immunohistochemistry assays were commercially purchased: anti-USP27 (Zen Bioscience, 613304, Chengdu, China); anti-SETD3 (Zen Bioscience, 508071), anti-Flag (Zen Bioscience, 390002), anti-myc (Zen Bioscience, 390003,); anti-HA (Beyotime, AF0039, Shanghai, China,) and anti-glyceraldehyde-3-phosphate dehydrogenase (GAPDH) (Beyotime, AF0006). HRP-conjugated secondary antibodies to mouse (Beyotime, A2016), or rabbit (Beyotime, A0208). Cycloheximide (CHX), protease inhibitor, and Cell Counting Kit-8 were purchased from MedChem Express (HY-K0301, Monmouth Junction, NJ, USA). MG132 was obtained from Sigma-Aldrich.

### Cell culture and transfection

Cell lines human liver cancer Hep3B and MHCC97H cells were purchased from Procell Life Science & Technology Co. Ltd. (Wuhan, China) and the Cell Bank of Chinese Academy of Sciences (Shanghai, China), respectively. Human embryonic kidney 293T (HEK293T) cells were gifted by Li Zhong (Chongqing University). HEK293T, Hep3B and MHCC97H cells were cultured in Dulbecco’s modified Eagle’s medium (DMEM) containing L-glutamine supplemented with 10% fetal bovine serum (FBS), and penicillin (100 U/ml)/streptomycin (100 μg/ml). Cells were grown in a humidified incubator at 37 °C in the presence of 5% CO_2_. Transfections were performed using Lipofectamine 2000 (Invitrogen; 11668-019) according to the manufacturer's instructions.

### Western blot and immunoprecipitation

The expression of proteins was examined as previously described [32]. Briefly, cells were lysed 48 h of post-transfection in buffer containing 50 Mm Tris–HCl (pH 8.0), 150 mM NaCl, 1% NP-40, 2 mM EDTA, 0.1% SDS, and protease inhibitors. After centrifugation at 13,000 rpm for 10 min, protein concentrations were measured using the BCA protein assay kit according to the manufacturer’s protocol. Lysates were separated by sodium dodecyl sulfate-polyacrylamide gel electrophoresis (SDS-PAGE) and then transferred to polyvinylidene difluoride (PVDF) membranes and immunoblotted with the desired antibodies. The lysates were immunoprecipitated overnight at 4 °C with 1 μg of the desired antibodies and 25 μl protein A-Sepharose (Santa Cruz, SC-2001). Subsequently, the precipitates were washed three times with cell lysis buffer, and the immune complexes were eluted with sample buffer containing 1% SDS for 5 min at 95 °C and analyzed by SDS-PAGE.

### Glutathione S-transferase (GST) pull-down experiments

GST pull-down experiments were conducted as previously describe [33]. Briefly, GST or GST-USP27 fusion constructs were expressed in *Escherichia coli* BL21 cells, and crude bacterial lysates were prepared via sonication in cold phosphate-buffered saline (PBS) in the presence of a protease inhibitor Phenylmethylsulfonyl fluoride (PMSF). Add 30 μl of the 50% BeyoGold™ GST-tag Purification Resins (Beyotime, P2250), and shake it slowly on a shaker at 4℃ for 4 h to fully bind the target protein with GST tag. Resins were eluted by an excessive quantity of reduced glutathione (GSH). Then, the supernatant was collected to obtain purified GST or GST-USP27 fusion protein. Flag-SETD3 fusion protein was then purified from cell lysates using anti-Flag affinity gel (Beyotime, P2271) and 3 × Flag peptide (Beyotime, P9801-1 mg) according to the manufacturer’s protocol. In brief, the cell lysates were incubated overnight at 4℃ with 30 μl of anti-Flag affinity to pull down the Flag-SETD3 protein. Beads were washed three times with tris-buffered saline (TBS). Then added 150 μl of 3 × Flag peptide eluent (150 μg/ml) to each sample, and incubated at 4℃ for 4 h. During this step, 3 × Flag peptide will compete for the binding sites on agarose beads, releasing the FLAG-tagged target protein into the eluate. Spin samples in a microcentrifuge at 6000×*g* for 30 s to obtain supernatant containing Flag-SETD3 protein. Use Coomassie staining to determine the quality of purified proteins. For pull-down assay, 5 μg of GST tag-fused USP27 protein was mixed with 5 μg of purified Flag tag-fused SETD3 protein, and then incubated with GST binding resins at 4℃ for 8 h. Resins were washed with binding buffer three times, resuspended in 30 μl of 2 × SDS-PAGE loading buffer, and resolved on 10% gels. Protein bands were detected with specific antibodies using western blot.

#### Proximity ligation assay (PLA)

PLA was proceeded as previously described [34]. Briefly, HEK293T cells were transfected with myc-USP27 and Flag-SETD3. Cells were cotransfected with myc-USP27 together with empty Flag vector served as controls. PLA were performed using Duolink® In Situ Red Starter Kit Mouse/Rabbit (Sigma-Aldrich, DUO92101) and anti-Flag tag rabbit (BBI Life Sciences, D110005-0200)/anti-myc tag mouse (Zen Bioscience, 390003) antibodies, according to the manufacturer’s instructions.

### Ubiquitination analysis

Ubiquitination assay was performed as previously described [35]. In brief, cells were lysed with a high concentration of SDS lysis buffer, denatured, immunoprecipitated with appropriate antibodies, and subjected to immunoblotting.

### shRNA knockdown and cycloheximide chase analysis

To knockdown USP27, lentiviral vector [36] was obtained from GeneCopoeia (Rockville, MD) and Supernatants containing viruses were packed in 293 cells. When growing to 60–80% confluence, Hep3B were infected with viral supernatants and 5 μg/ml puromycin was added to select the stable cells [37]. Nonspecific shRNA was used as a control. The sequences were shown as follows: for shCtrl, 5′-GATCCGTTCTCCGAACGTGTCACGTAATTCAAGAGATTACGTGACACGTTCGGAGAATTTTTTC-3ʹ (forward) and 5′-AATTGAAAAAATTCTCCGAACGTGTCACGTAATCTCTTGAATTACGTGACACGTTCGGAGAACG-3ʹ (reverse); for shUSP27-1, 5ʹ-CCGGGACGCCGTTTATGGCCTCAAGTAAACTCGAGTTTACTTGAGGCCATAAACGGCGTCTTTTT-3ʹ (forward) and 5ʹ-AATTAAAAAGACGCCGTTTATGGCCTCAAGTAAA CTCGAGTTTACTTGAGGCCATAAACGGCGTC-3ʹ(reverse); for shUSP27-2, 5ʹ-CCGGCACTGGTACATATCCTATATTCTCGAGAATATAGGATATGTACCAGTGTTTTTG-3′ (forward) and 5′-AATTCAAAAACACTGGTACATATCCTATATTCTCGAGAATATAGGATATGTACCAGTG-3′ (reverse); for shSETD3-1, 5′-CCGGGATGTCTTCAGCCAGTATAAACTCGAGTTTATACTGGCTGAAGACATCTTTTTG-3ʹ (forward) and 5′-AATTCAAAAAGATGTCTTCAGCCAGTATAAACTCGAGTTTATACTGGCTGAAGACATC-3ʹ (reverse); and for shSETD3-2, 5′-CCGGGAAGAAGATGAAGTTCGGTATCTCGAGATACCGAACTTCATCTTCTTCTTTTTG-3ʹ (forward) and 5′-AATTCAAAAAGAAGAAGATGAAGTTCGGTATCTCGAGATACCGAACTTCATCTTCTTC-3ʹ (reverse).

293T or hepatocellular carcinoma cells were transfected with the indicated constructs and grown for 24 h. CHX (100 μg/ml) was added to inhibit protein synthesis. Equal numbers of cells were collected at the different time points. The protein level of SETD3 in the samples was examined by immunoblotting as described in the text and corresponding figure legends.

### RNA isolation and qRT-PCR

Total RNA was collected using TRIzol reagent (TaKaRa, Japan) according to the manufacturer’s protocol. cDNA was reverse transcribed from 1 μg of total RNA using a reverse transcription kit (TaKaRa, Japan). β-actin was used to normalize sample. qPCR primers: USP27 forward: 5ʹ-TCACCCACACGCCGATACT-3ʹ, Reverse: 5ʹ-CCAGACACAACTCGGGACTC-3ʹ; SETD3 forward: 5’-CAACCTGGAAGATGA CCGCTGT-3’, Reverse, 5’-CACTGTGGATCACAAACTCTGCG-3’; β-actin forward: 5ʹ-CATGTACGTTGCTATCCAGGC-3ʹ, Reverse: 5ʹ-CTCCTTAATGTCACGCACGAT-3ʹ. Amplification was performed according to the instructions for the 2 × SYBR Green Master Mix (BIO-RAD) using a BIO-RAD C1000™ Thermal Cycler. PCR was performed at 95 °C for 30 s of initial denaturation and then at 95 °C for 30 s of denaturation, 60 °C for 30 s of annealing/extension, 40 cycles. Relative expression levels were calculated using the 2^−ΔΔCt^ method for qRT-PCR.

### CCK-8 assay and colony formation assays

Cell proliferation and colony formation assays were conducted as described previously [32, 38]. 1 × 10^3^ cells were seeded into 96-well plates in triplicate, and the Cell Counting Kit-8 (CCK-8) was used to monitor the cell proliferation rate continuously until 96 h. CCK-8 was added to each well and the cells were incubated for 2 h at 37 °C. Subsequently, the absorbance value was read at 450 nm. For the colony formation assay, 1 × 10^3^ cells were seeded in six-well plates to form colonies. After 2–3 weeks, colonies were fixed with 4% paraformaldehyde for 10 min, stained with crystal violet for 20 min, photographed, and counted with Image J software.

### EdU staining

EdU cell proliferation staining was performed as described previously using an EdU kit (BeyoClick™ EdU Cell Proliferation Kit with Alexa Fluor 488, C0071S, Beyotime, China) [39]. Briefly, Hep3B cells (1 × 10^4^ cells/well) were cultured on round coverslips in 12-well plates for 12 h. Subsequently, cells were incubated with EdU for 2 h, fixed with 4% paraformaldehyde for 15 min, and permeated with 0.3% Triton X-100 for another 15 min. The cells were incubated with the Click Reaction Mixture for 30 min at room temperature in a dark place and then incubated with Hoechst 33342 for 10 min to counterstain the nucleus.

### Tumorigenesis in nude mice

BALB/c nude mice (4 weeks old, female) were housed and maintained under special pathogen-free (SPF) condition. All animal experimental procedures were approved by the Animal Care and Use Committee of Chongqing University. Different cell lines and control cells were trypsinized into single cell suspension and resuspended in PBS with a concentration of 5 × 10^6^/100 μl. Mice were randomly divided into groups (*n* = 6 for each group) and injected subcutaneously in the right flanks with 100 μl of the cell suspensions. Tumors were measured every 3 days with a slide caliper and tumor volume was calculated by the formula: volume (mm^3^) = 0.5 × length × width^2^. After 27 days, mice were sacrificed and tumors were dissected, photographed and weighted.

### Ki67 staining

Ki67 staining carried out as described previously [40]. Briefly, cells (5 × 10^3^/well) were grown in 12-well plates for 12 h, then rinsed with PBS, fixed in 4% paraformaldehyde for 15 min, and permeabilized with 0.1% Triton X-100 for 15 min. After blocking with 10% normal goat serum in PBS for 30 min, cells were incubated with a rabbit anti-Ki67 antibody (dilution 1:200, AF1738, Beyotime, China) overnight at 4 °C followed by an incubation with a fluorescein isothiocyanate (FITC)-conjugated goat anti-rabbit IgG (green, dilution 1:200, Sangon Biotech, China) for 1 h. Nucleus were counterstained with DAPI (blue, Servicebio, China).

For tumors from nude mice model, Ki67 were detected by standard immunohistochemistry protocols. Tissue specimens were deparaffinized, hydrated and boiled in EDTA buffer (pH 8.0) for 3 min for antigen retrieval. After treated with 3% H_2_O_2_ for 10 min, slides were blocked with 10% normal goat serum for 30 min, and then incubated with a rabbit anti-Ki67 antibody (dilution 1:200, AF1738, Beyotime, China) overnight at 4 °C, followed by an incubation with HRP-conjugated secondary antibody (dilution 1:100, A0208, Beyotime, China) for 1 h. Then, the signal was developed by in 3,3-diaminobenzidine (DAB) solution for 5 min and the nucleus were counterstained with hematoxylin (blue).

### Wound-healing assay

Wound-healing assay was conducted as previously described. In brief, a single scratch wound was created using a sterile 10 μL plastic pipette tip across the cell surface. The area of a defined region within the scratch was measured using Image J software. The extent to which the wound had closed over 24 h was calculated and expressed as a percentage of the difference between time points 0 and 24 h.

### Transwell migration assay

Transwell migration assay was performed as described previously [41]. In brief, 1 × 10^4^ cells in serum-free medium were seeded into 8.0 μm pore transwell filters (Falcon, Corning). DMEM containing 10% FBS were added to the bottom chamber as attractants. After incubation for 24 h, the cells on the top side of the filter were removed by washing. The attached cells at the bottom of the filter were fixed with 4% paraformaldehyde and were stained with crystal violet. Then, the number of invaded cells was examined with Nikon microscope at 100 × magnification. 3 random fields of each group were photographed for counting purposes and the average number of migrated cells was used as a measure of migration capacity.

### Statistical analysis

The data were presented as the means ± standard error of mean (SEM) from triple independent experiments. Data were analyzed with Prism 8.0 (GraphPad Software, San Diego, CA). P value less than 0.05 was considered statistically significant.

## Supplementary Information

Below is the link to the electronic supplementary material.

Supplementary file1 (DOCX 1031 KB)

## Data Availability

All data generated or analyzed during this study are included in this published article. Data and material will be made available on reasonable request.
