# Supplementary material for: Stabilization of SETD3 by deubiquitinase USP27 enhances cell proliferation and hepatocellular carcinoma progression
Source: Cell Mol Life Sci. 2022 Jan 12;79(1):70. doi: 10.1007/s00018-021-04118-9 (PMC8752572; doi:10.1007/s00018-021-04118-9)
Supplement: Supplementary file 1 — Supplementary file1 (DOCX 1031 KB) [file 18_2021_4118_MOESM1_ESM.docx]

**
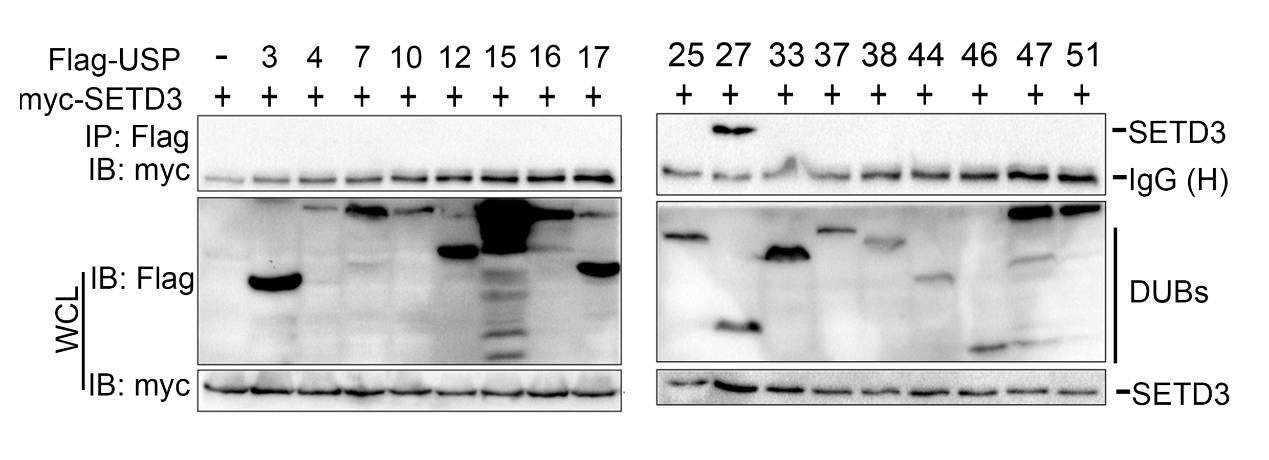
**

**Fig. S1** Identification of SETD3 interaction proteins by immunoprecipitation assay. The interactions between SETD3 and deubiquitinases (DUBs) were detected in HEK293T(293T) cells. 293T cells were transfected with SETD3 and each of DUBs and immunoprecipitation and immunoblot were performed with corresponding antibodies.

**
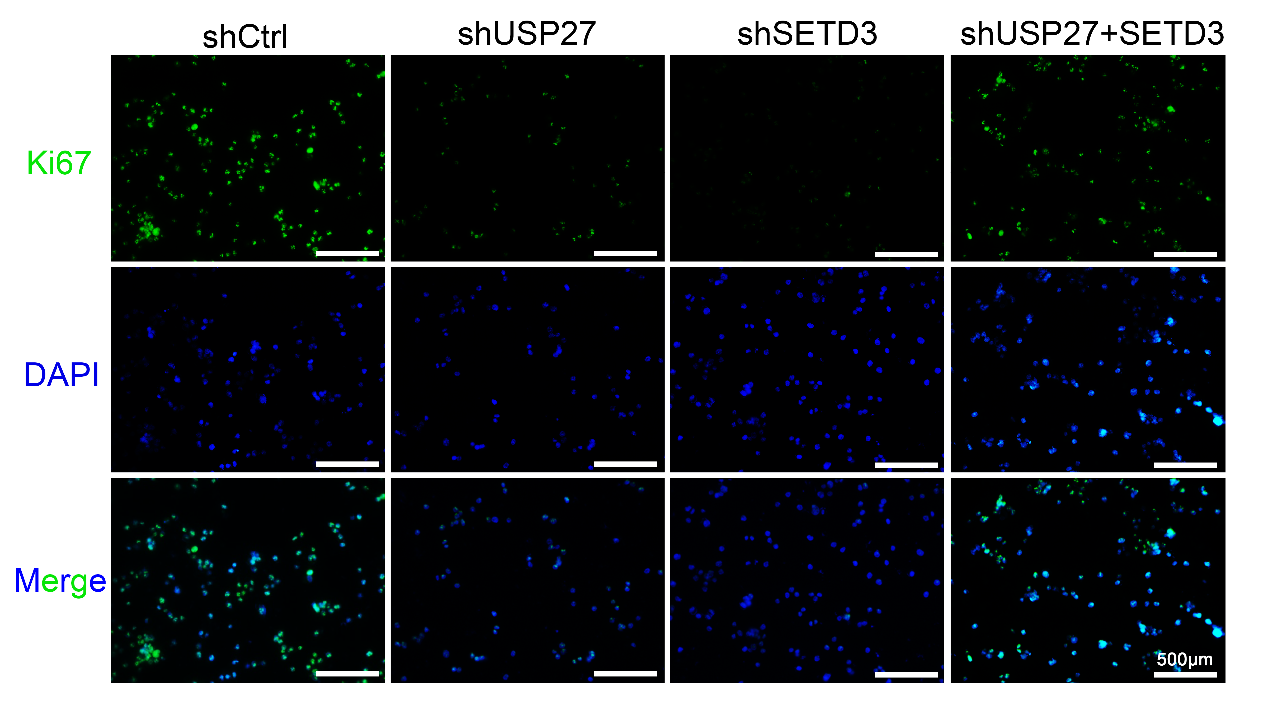
**

**Fig. S2** Ki67 staining showed that Ki67-positive proliferating cells were significantly decreased upon USP27 or SETD3 knockdown in Hep3B cells, while overexpression of SETD3 in USP27 knockdown cells could restore cell viability (Scale bar, 500 μm).


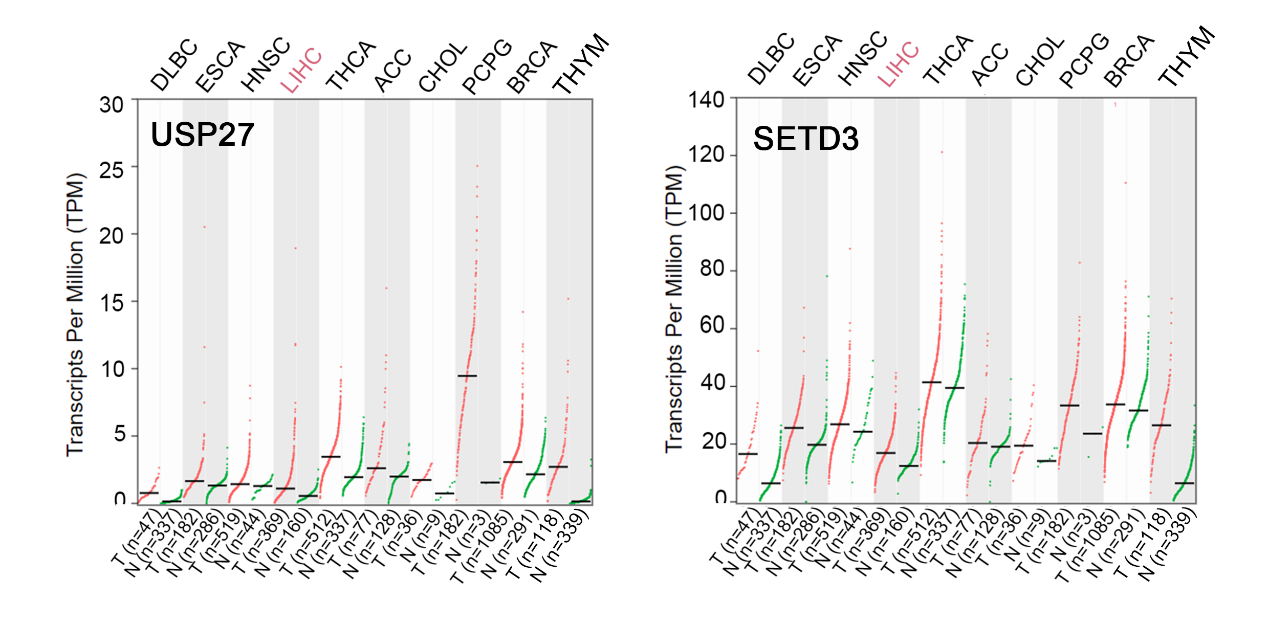


**Fig. S3** Relative USP27 and SETD3 expression in 10 cancers and their corresponding normal tissues. The expression data were analyzed by GEPIA2 server, integrated with TCGA and GTEx databases and the threshold was set as log_2_FC > 1, *p* < 0.01. Each red dot in the diagram represents a tumor tissue sample and each green dot represents a normal tissue sample. T: tumor, N: normal tissue, n: number. DLBC: lymphoid neoplasm diffuse large B-cell lymphoma; ESCA: esophageal carcinoma; HNSC: head and neck squamous cell carcinoma; LIHC: liver hepatocellular carcinoma; THCA: thyroid carcinoma; ACC: Adrenocortical carcinoma; CHOL: cholangiocarcinoma; PCPG: pheochromocytoma and paraganglioma; BRCA, breast invasive carcinoma; THYM: thymoma.
